# Use of commercial infant cereals as complementary food in infants and young children in Ghana

**DOI:** 10.1186/s40795-017-0191-x

**Published:** 2017-08-31

**Authors:** Abdul-Razak Abizari, Zakari Ali, Collins Nana Essah, Patience Agyeiwaa, Margaret Amaniampong

**Affiliations:** Department of Nutritional Sciences, School of Allied Health Sciences, University for Development Studies, P.O. Box TL 1883, Tamale, Ghana

**Keywords:** Commercial infant cereals, Micronutrients, Young children, Ghana

## Abstract

**Background:**

Typical complementary foods in developing countries are often inadequate in multiple micronutrients. One way of preventing micronutrient deficiency among infants and young children (IYC) is to feed them a variety of nutrient dense foods. The use of commercial infant cereals (CIC) as complementary food is increasing in Ghana. However, it is unknown whether giving CIC may discourage inclusion of other locally available and nutritious foods as mothers may hold the notion that CIC is superior. This study therefore examined the use of CIC as complementary food, the micronutrient intake from CIC and reasons for its use.

**Methods:**

A cross-sectional study was conducted involving mothers with IYC aged 6-23 months who give CIC as complementary food. Questionnaires were administered to estimate the usual daily frequencies and quantities of CIC. Micronutrient intakes from CIC were calculated. We also calculated the proportion of the recommended nutrient intake (RNI) contributed by micronutrients from CIC.

**Results:**

Of the 384 children sampled, 64.6% were aged 13-23 months and 50.3% were females. More than half of the mothers earned income through trading (55.2%) and nearly one quarter of them had senior high school education (24.7%). Majority of the children consumed 3–4 tablespoons (52.3%) of CIC in a feeding moment. Younger children (6–12 months) were served CIC more frequent than older children (13–23 months). The mean ± SD of micronutrients from CIC were 6.76 ± 2.67 mg, 4.79 ± 1.70 mg, 384.12 ± 139.69 mg, 266.70 ± 100.74 mg and 69.11 ± 24.65 μg for the minerals Fe, Zn, Ca, P and I respectively. The vitamin intakes were: 337.17 ± 119.70 μg, 42.88 ± 15.28 mg, 0.84 ± 0.31 μg, 34.24 ± 12.22 μg and 2.70 ± 1.03 mg for vitamin A, vitamin C, vitamin B12, folic acid and niacin respectively. Micronutrients from CIC consumption contributed to at least 70% of the RNIs for Fe, Zn, Ca, I, vitamin A, vitamin C and vitamin B12. Mothers believed that CIC alone could meet the nutritional needs of children and ensure optimal growth and health.

**Conclusion:**

The results of present study show that use of CIC as complementary food was high among mothers with IYC 6–12 months. Mothers believed that CIC was nutritionally adequate for their children and did not see the need to include or enrich other locally available complementary foods. Mothers should be educated on the need to include other foods with CIC to increase the dietary quality of IYC.

## Background

Adequate nutrition during infancy and early childhood is fundamental to the development of a child’s full potential [1]. Micronutrient deficiency is of public health concern in developing and developed countries and affects about 2 billion people [2]. Given the increased nutrient demands of young children, they are one of the population groups most vulnerable to micronutrient deficiencies [3]. Childhood deficiency in zinc and vitamin A is linked to child deaths [4]. The combined effect of iodine deficiency and stunted growth also affect a child’s development potential [4].

Breast milk alone can totally satisfy the nutritional requirements of infants within the first 6 months after birth [5]. After this period, breast milk becomes inadequate for optimal growth and development and complementary foods are introduced to increase energy and nutrient intake [6]. Breastfeeding is recommended to continue along with complementary feeding until a child’s second birthday or beyond [6]. Complementary feeding is important, considering the rapid mental and physical developments that take place in this age group which occur with a particularly high need for micronutrient intake [7]. Foods for complementary feeding therefore need to be more nutrient-rich than the foods typically consumed by other age groups in the household [8]. Complementary foods are regarded transitional foods and usually include local and industrially processed foods [9] including CIC [10].

Typical complementary foods in developing countries are often inadequate in multiple micronutrients especially iron and zinc [8, 11, 12]. To ensure adequate nutrition in these children, the World Health Organization recommends age appropriate complementary feeding that meets both frequency and diversity [6, 13]. It is recommended that IYC consume from at least four out of seven core food groups in a day to ensure adequate nutrient intake [13]. Dietary diversity is shown to be associated with adequate micronutrient intake in different developing country settings [14, 15]. Even though CIC are fortified with some micronutrients [16], mostly iron [17], calcium, zinc, phosphorus, thiamine, riboflavin and niacin [18] which are key to the growth and development of IYC, consumption these foods alone may not meet daily recommended needs of IYC [19].

CICs are increasingly becoming popular as complementary food in Ghana, especially among urban mothers [20, 21]. As mothers may hold the notion that CIC are superior to local complementary foods, CIC use may discourage inclusion of other locally available and nutritious foods and reducing dietary diversity of IYC. CIC usage and consumption levels among IYC, the reasons mothers may prefer feeding their IYC with CIC instead of using locally available, nutritious and relatively cheap alternatives are unknown. This study therefore examined the use of CIC as complementary food, the consumption levels among IYC, the micronutrient content of commonly used CIC and reasons mothers may prefer CIC to enriching local complementary foods.

## Methods

### Study area

This study was conducted in Tamale, the administrative capital of the Northern region of Ghana. Tamale is one of the largest cities in Ghana and a metropolitan with an estimated land size of 646.9 sq. km located in the central part of the Region [22]. The total population size of the metropolis is estimated at 233,252 which is about 9.4% of the region’s population. The population is largely urban (80.8%) with a few living in rural localities (19.1%) of the metropolis. Women of reproductive age (15–49 years) in the metropolis make up about 23.8% of the population and children aged 0–4 years are 14% of the total population. The General Fertility Rate is 79.9 births per 1000 women aged 15–49 years. There are four major markets in the Metropolis namely: Central Market, Aboabo, Kukuo and Lamashegu. In addition to these, there are satellite markets in large suburbs of the Metropolis. The Central market comprises of mini shops and stalls. There are also supermarkets in the city where commercial infant cereals are purchased [23].

### Design, target population and sampling

A descriptive cross sectional study design was used in the present study. The target population was mothers with infants and young children 6–23 months in Tamale Metropolis. This study included only mothers who had children 6–23 months and used commercial infant cereal as part of complementary foods given to the child.

Using an alpha of 1.96 at 95% confidence interval with a permitted 5% margin of error and an assumed 50% prevalence of micronutrient adequacy from commercial infant cereals in the Northern region, the required sample size was 384. Data were collected mainly from five urban suburbs (Aboabo, Lamashegu, Vitting, Sakasaka, and Nyohini) within the Tamale metropolis through visits to selected households. Mothers using CIC as complementary food in selected communities were first identified at child welfare clinics and followed home to complete informed consent and research questionnaire. Recruitment at child welfare clinics continued until the required sample size was met.

### Data collection tools and procedures

Semi structured questionnaires were used for data collection. Questionnaire administration was done through face-to-face interviews in the language the respondent understood. The questionnaire elicited information about maternal and child demographic characteristics, use of commercial infant cereal, preferred brand of infant cereal, number of times CIC is served in a day, and quantity of CIC fed to child in a feeding moment.

### Estimation of quantity of infant cereal consumed in a day

The number of times CIC was consumed during the preceding 24 h and the estimated number of tablespoons of CIC consumed by children per feeding moment were determined by recall. For mothers who gave sachets (50 g) of CIC, their equivalents in household tablespoons were estimated. The total tablespoons of CIC consumed in a day by children was calculated by multiplying the number of times CIC was consumed in a day by the average (usual) number of tablespoons of CIC consumed per feeding moment.

From a subgroup of 20 mothers, we estimated the quantity (in grams) of CIC equivalent to a typical tablespoon given by mothers. We used the two most preferred brands of CIC (Cerelac and Yumvita) for this estimation. Mothers were presented their preferred brand and asked to use their usual household tablespoon to fetch a usual tablespoonful of CIC for their infant or young child. Each mother or caregiver gave two scoops of a usual tablespoonful of CIC. Each scoop was weighed with an electronic scale (Citizen CY 360, precise to 20 mg) and an average of the two was taken as the estimated weight of the usual tablespoonful for each brand of CIC. The average weight of a typical tablespoonful of CIC offered to children was calculated from the averages of the two most preferred brands.

### Estimation of daily micronutrient intake from CIC

Daily micronutrient intake of children from CIC was estimated by determining the micronutrient content of a typical tablespoon of the preferred brand of CIC and the total tablespoons consumed in a day. The micronutrient content of the preferred brand and flavour of CIC was listed per 100 g of the product by the manufacturer. The micronutrient content of a typical tablespoon of mother’s preferred brand and flavour of CIC was calculated by proportion. Vitamin A content of CIC was stated in international units (IU), these were converted to micrograms (μg) by multiplying by 0.3 [24]. The amount of micronutrients consumed in a day by IYC from CIC was calculated by multiplying the amount of micronutrient in a typical tablespoon of the preferred brand and flavour by the total tablespoons of CIC consumed in a day. We made the following assumptions for the estimation of daily micronutrient intake from CIC: (i) children completely consumed the quantity of CIC prepared for them per eating moment (ii) the micronutrient content of different brands (as listed by the CIC manufacturers) are reasonably stable. Probabilities of inadequacy of the micronutrients were not calculated as at least two days of repeated dietary recalls are required [24]. We calculated the proportion of the RNI contributed by micronutrients from CIC. We used RNIs provided by FAO/WHO [25] and assumed 12% and moderate bioavailability for iron and zinc respectively.

### Data analysis

Data was entered, cleaned and analyzed using IBM SPSS (version 21). Continuous data were presented as means and standard deviations while categorical data were presented as frequencies and percentages.

## Results

### Socio-demographic characteristics of respondents

The mean ± SD age of mothers in this study was 28.7 **±** 4.7 years and four in ten mothers were within the age range 25–29 years (40.1%). Two-thirds of the mothers/caregivers have attained at least junior high school education. Majority of the mothers were married (92.2%), practiced Islamic religion (75.3%), earned income through trading (55.2%) and were from the Dagomba ethnic group (58.1%). The average child in this study was a little above the first birthday (14.9 **±** 4.8 months) with more than six in ten aged 13–23 months (64.6%). The proportion of female children in the sample was a little more than half (50.3%) (Table 1).

**Table 1:** Demographic characteristics of sample

Characteristics	Mean ± SD	Frequency	Percentage
Child characteristics			
Sex			
Male		191	49.7
Female		193	50.3
Mean age (months)	14.9 ± 4.8		
Age group (months)			
6–12		136	35.4
13–23		248	64.6
Maternal characteristics			
Mean age of mothers (years)	28.7 ± 4.7		
Age group of mothers (years)			
< 20		2	0.5
20–24		65	16.9
25–29		154	40.1
30–34		118	30.7
35–39		35	9.1
40+		10	2.6
Education			
None		77	20.1
Primary		50	13.0
JHS		90	23.4
SHS		95	24.7
Tertiary		72	18.8
Marital status			
Married		354	92.2
Currently unmarried		4	1.0
Religion			
Islam		289	75.3
Christian		93	24.2
Other		2	0.5
Ethnicity			
Dagomba		223	58.1
Gonja		51	13.3
Akan		50	13.0
Other		60	15.6
Occupation			
Farmer		14	3.6
Trader		212	55.2
Civil servant		69	18.0
Unemployed		38	9.9
Other		51	13.3

### Knowledge and opinion of respondents on commercial infant cereal utilization

Table 2 presents information on the knowledge and opinions of mothers on the use of CIC. The majority of the mothers had information about CIC from the media (radio and television advertisements) (50.3%) and knew at least two brands (57.0%) of CIC on the Ghanaian market. Although the majority of mothers knew more than one brand of CIC, more than nine in ten mothers purchased Cerelac brand (Nestle). Majority of mothers purchased CIC packaged in tin containers (79.9%) rather than single use packs. Forty-six percent of mothers held more than one reason for using CIC as complementary food for their children. Some of the specific reasons given for utilization of CIC include: CIC help in infant growth and development (29.7%), it provides sufficient nutrients for infants and young children (9.4%) and easy to prepare (4.2%).

**Table 2:** Knowledge on commercial infant cereal and maternal opinions on its use

Item	Frequency	Percentage
Source of information on CIC		
Media (radio and television)	193	50.3
Health worker	122	31.8
Friends	43	11.2
Others	26	6.8
Brand(s) of CICs known		
Cerelac	157	40.9
Motherluck	5	1.3
Yumvita	3	0.8
Two or more of the brands	219	57.0
Brand of CIC used for child		
Cerelac	350	91.1
Motherluck	4	1.0
Yumvita	30	7.8
Package of CIC used		
Tin (400 g)	307	79.9
Sachet (50 g)	77	20.1
Mothers’ opinion on CIC as complementary food		
Helps in infants growth and development	114	29.7
Provides sufficient nutrient for infants and young children	36	9.4
Infants and young children prefer it to infant formula	20	5.2
Easy to prepare	16	4.2
It prevents diseases in infants and young children	8	2.1
Two or more of the opinion	177	46.1
Others	12	3.1
Duration of CIC use as complementary food		
At least 1 month	18	4.7
2–6 months	127	33.1
7–12 months	142	37.0
More than 12 months	97	25.3

### Daily intake of commercial infant cereal by infants and young children

Children ate between 1 and 5 tablespoons of CIC in a feeding moment. Overall, most of them ate 3–4 tablespoons (52.3%) of CIC in a feeding moment. The number of tablespoons of CIC consumed by children in a serving differed by age group. For instance, while the majority of children aged 6–12 months ate at least 2 tablespoons (80.9%), the majority of those aged 13–23 months ate 3–4 tablespoons (70.6%) in a feeding moment. In a day, children were served CIC between 1 and 3 times. A little above six in ten children were served two times (62.0%) of CIC in a day. Younger children (6–12 months) were served with CIC more frequent than older children (13–23 months); while the majority of children 6–12 months were served three times (55.9%) of CIC in the day, the children aged 13–23 months were served two times (73.8%) of CIC in the day. The estimated average weight of a usual tablespoon of CIC given to children was ~15.0 ± 0.5 g (Table 3).

**Table 3:** Daily intake of commercial infant cereal

Variable	Frequency (%)		
Age groups (months)	6–12	13–23	All
n	136	248	384
Tablespoons per serving			
1–2	110 (80.9)	69 (27.8)	179 (46.6)
3–4	26 (19.1)	175 (70.6)	179 (52.3)
≥ 5	0 (0.0)	4 (1.6)	4 (1.0)
Mean tablespoons ± SD	2.15 ± 0.54	2.95 ± 0.73	2.67 ± 0.77
Frequency CIC is served per day			
Once	5 (3.7)	29 (11.7)	34 (8.9)
Two times	55 (40.4)	183 (73.8)	238 (62.0)
Three times	76 (55.9)	36 (14.5)	112 (29.2)
Weight of tablespoon of CIC (g)			Mean ± SD
n			20
Cerelac			14.6 ± 3.0
Yumvita			15.3 ± 3.4
Both			15.0 ± 0.5

### Micronutrient content and intake of commercial infant cereals

Table 4 presents the micronutrient content per 100 g of the most preferred brands and flavour, and per tablespoon of CIC consumed by children in this study. The micronutrient content of CIC (as stated by the manufacturer) varied greatly among different brands of CIC. For example, while the iron content of Yumvita was 12 mg per 100 g, the iron content of Cerelac and Motherluc were 7.5 mg per 100 g and 2.6 mg per 100 g respectively.

**Table 4:** Micronutrient content of infant cereals per 100 g and per tablespoon

Micronutrient	Brand of infant cereal					
	Cerelac (wheat)		Yumvita (wheat)		Motherluc wheat cereal	
Content	Per 100 g	Per tablespoon	Per 100 g	Per tablespoon	Per 100 g	Per tablespoon
Iron (mg)	7.5	1.125	12	1.80	2.6	0.39
Zinc (mg)	5.5	0.825	6	0.90	ns	-
Phosphorus (mg)	320	48.0	200	30.0	84	12.6
Iodine (μg)	80	12.0	ns	-	ns	-
Vitamin A (μg RAE)	390	58.50	390	58.5	ns	-
Vitamin C (mg)	50	7.50	45	6.75	ns	-
Vitamin B12 (μg)	1	0.15	0.6	0.09	ns	-
Folic acid (μg)	40	6.00	35	5.25	ns	-
Niacin (mg)	3	0.45	4.5	0.675	ns	-

1 tablespoon = 15.0 g

*ns* not stated

The mean ± SD of micronutrient intake from CIC were 6.76 ± 2.67 mg, 4.79 ± 1.70 mg, 384.12 ± 139.69 mg, 266.70 ± 100.74 mg and 69.11 ± 24.65 (μg) for the minerals; Iron, Zinc, Calcium, Phosphorus and Iodine respectively. The vitamin intakes were; vitamin A (337.17 ± 119.70 μg), vitamin C (42.88 ± 15.28 mg), vitamin B12 (0.84 ± 0.31 μg), folic acid (34.24 ± 12.22 μg) and niacin (2.70 ± 1.03 mg). Micronutrients from CIC consumption contributed to at least 70% of the RNIs for Iron, Zinc, Calcium, Iodine, vitamin A, vitamin C and vitamin B12 (Table 5).

**Table 5:** Estimated daily micronutrient intake from commercial infant cereals

Micronutrient	n	6–12 months	% RNI	13–23 months	% RNI	All	% RNI
Minerals (mean ± SD)							
Iron (mg)	384	6.44 ± 2.62	83.61	6.93 ± 2.68	114.43	6.76 ± 2.67	122.89
Zinc (mg)	380	4.56 ± 1.68	111.12	4.92 ± 1.71	120.03	4.79 ± 1.70	116.84
Calcium (mg)	384	368.48 ± 134.87	92.12	392.70 ± 141.80	78.54	384.12 ± 139.69	83.35
Phosphorus (mg)	384	256.17 ± 96.80	na	272.47 ± 102.58	na	266.70 ± 100.74	na
Iodine (μg)	350	65.40 ± 24.21	72.67	71.22 ± 24.71	79.13	69.11 ± 24.65	76.79
Vitamins (mean ± SD)							
Vitamin A (μg RAE)	380	320.89 ± 117.55	80.22	346.24 ± 120.17	86.56	337.17 ± 119.70	84.29
Vitamin C (mg)	380	40.84 ± 14.96	136.14	44.02 ± 15.37	146.73	42.88 ± 15.28	142.94
Vitamin B12 (μg)	380	0.80 ± 0.30	114.15	0.86 ± 0.32	95.35	0.84 ± 0.31	102.08
Folic acid (μg)	380	32.61 ± 11.96	40.77	35.14 ± 12.30	23.43	34.24 ± 12.22	29.63
Niacin mg	380	2.56 ± 1.01	63.96	2.77 ± 1.03	46.26	2.70 ± 1.03	52.60

*na* RNI not available for this nutrient

## Discussion

This study sought to determine the use of CIC as complementary food, the consumption levels among different age groups of IYC, the micronutrient content of commonly used CIC and reasons mothers prefer CIC to enriching local complementary foods. The main findings were that CIC use among mothers was high, younger children 6–12 months were more likely to depend entirely on CIC as complementary food but not those aged 13–23 months and mothers held various reasons and opinions (including; believing that CIC help in infant growth and development, provides sufficient nutrients for their children and easy to prepare) for depending on CIC as complementary food for their children instead of giving a diversified diet made of locally available and nutritious foods.

The high use of CIC as complementary food among mothers in this study is consistent with the findings of a study in urban Ghana among mothers giving complementary foods [20]. A recent survey of the market revealed a wide range of different CIC brands on the Ghanaian market [21], probably driven by a high demand. Mothers in this study believed that the use of CIC was good for child growth and development and was enough to meet the nutritional needs of their IYC. This is probably the reason for the high demand of CIC by mothers. With this notion that CIC could meet the nutritional needs of their children and promote growth, mothers may not see the need to include locally available and nutrient dense complementary foods which would improve dietary diversity and meet nutritional needs. Contrary to our findings, an earlier study reported convenience as a major influence for the use of CIC with health benefits as second.

The majority of mothers used Cerelac (Nestle Ghana limited) as their preferred brand of CIC for complementary feeding. The dominance of Cerelac as a preferred CIC in present study is consistent with earlier studies in Ghana [20, 21]. The wide use of this brand could be due to the variety of flavours it presents (maize, wheat, rice, millet, yummy fruits and fruits) and the longer years it has been on the Ghanaian market as well as a strong media marketing. With Cerelac, mothers can easily vary the flavours from time to time to suit child’s preference or provide variety, however, the most preferred flavour was wheat flavour. The other available brands did not have as many flavours. For instance, Yumvita (Promasidor Ghana limited) had only two flavours (wheat and maize & wheat) and Motherluc (Atona foods investments Ghana) was available as only wheat flavour. As mothers change these flavours, it might appear to them as though they are diversifying the diet for their children, but it is the same cereal based foods eaten on different days, usually as child becomes fed up with one flavour. This does not increase the diet diversity of children.

Children were served CIC between one and three times in a day. Similar frequency of infant cereal consumption has been reported among Ghanaian children [20]. Our data further reveals that younger children were fed CIC more frequent than older children. For younger children, high frequency of feeding CIC may mean fewer eating moments to offer other foods from the household. This makes these children almost entirely dependent on CIC for nutrients apart from breastmilk especially because mothers believe that CIC alone could satisfy the nutritional needs of their children. The lower frequency of feeding CIC among older children may be explained by the introduction and continuing feeding of household food. Further, it may be relatively more expensive to feed older children CIC more frequently because the quantity of intake may increase.

To meet micronutrient requirements of children taking complementary foods, fortified foods have been suggested to be included [26] and data from studies in different developing country settings have emphasized the importance of fortified foods in meeting micronutrient needs. For example, a study in Indonesia found that without inclusion of fortified foods (including infant cereal), it was unlikely for children especially those from low socioeconomic households to have adequate intakes of iron, zinc, niacin and thiamin [27]. Another study in rural Kenya has shown that dietary adequacy of iron, zinc and calcium are unlikely to be met by consumption of only local complementary foods and suggests fortified foods to ensure their adequacy [28]. Results from a study among South African children also show higher intake of iron, zinc, vitamin A, vitamin C, vitamin B6, vitamin B12 and niacin from complementary foods including CIC [29]. It is important to note that, CIC in these studies was given with other local complementary foods and therefore diet diversity was likely to be high and CIC added to the micronutrients from other foods consumed. For children to meet adequate intakes especially among the younger children who were less likely to consume other foods, mothers should be encouraged to include locally available and nutrient dense complementary foods to increase the dietary diversity and meet nutritional needs of IYC.

Cereals contain phytate [30] and polyphenols [31] which reduce the bioavailability of some micronutrients contained in them especially iron and zinc [32, 33]. Overcoming low bioavailability of iron in cereal based complementary foods is perhaps one of the greatest challenges with fortification of infant foods [34].The type of cereal grain used in preparation has little effect on iron bioavailability [35] and dephytinization studies have reported mixed findings [36, 37]. In spite of these, inclusion of enhancers of iron absorption such as ascorbic acid (vitamin C) has been successful in improving iron bioavailability from infant cereals [32, 38–40]. Estimated iron intake from CIC in present study is therefore likely to be absorbed as intake levels of vitamin C from CIC was appreciably high.

The interpretation of the findings in this study need to be made while keeping some limitations in mind. First, we assumed that children ate all portions that were prepared for them at each eating moment, but may not always be the case. There may be leftovers but our estimations of consumption and subsequent calculations did not account for that. If there were many instances of leftover then our estimations of micronutrient intake could be overestimated. We however believe that over time caregivers are able to serve quantities that do not always result in leftovers.

Secondly, we used the nutrient values specified by CIC manufacturers which may not always be entirely correct. Variations between manufacturers’ specified values on products and results of independent analyses of product samples have been reported [41, 42]. If the micronutrient contents specified by infant cereal manufacturers were over reported, our estimations of intake could also be overestimated. However, micronutrient content variations between manufacturers’ values and analyzed values are not always adverse. For instance, there were no adverse variations in the iodine content of different brands of bouillon cubes in Ghanaian markets between manufacturer values and analyzed values [43]. Moreover, manufacturer nutrient information could be used to build food composition databases [44], suggesting that they could be relied upon for nutrient assessment of foods.

Further studies are warranted to compare micronutrient adequacy of children who consume CIC and those who rely only on family foods and explore the effect of CIC use on recommended infant feeding indicators as present study did not include IYC who receive only family foods.

In spite of these limitations, our results have shed ample light on the use and consumption levels of CIC as complementary food among different age groups of children in Ghana.

## Conclusion

The results of present study show that use of CIC as complementary food was high among mothers with IYC 6–12 months. Mothers believed that CIC was nutritionally adequate for their children and did not see the need to include or enrich other locally available complementary foods. Mothers who offer CIC should be educated on the need to include other locally available and nutrient dense foods in the diets of IYC to ensure nutrient adequacy.

## Abbreviations

CIC: Commercial infant cereal
IYC: Infants and young children
RNI: Recommended nutrient intake
WHO: World Health Organization
